# The Syndromes of Thrombotic Microangiopathy: A Critical Appraisal on Complement Dysregulation

**DOI:** 10.3390/jcm10143034

**Published:** 2021-07-08

**Authors:** Sjoerd A. M. E. G. Timmermans, Pieter van Paassen

**Affiliations:** 1 Department Nephrology and Clinical Immunology, Maastricht University Medical Center, 6229 HX Maastricht, The Netherlands; p.vanpaassen@maastrichtuniversity.nl; 2 Department Biochemistry, Cardiovascular Research Institute Maastricht (CARIM), 6229 HX Maastricht, The Netherlands

**Keywords:** thrombotic microangiopathy, atypical hemolytic uremic syndrome, complement, hypertensive emergency, pregnancy, kidney transplantation, eculizumab

## Abstract

Thrombotic microangiopathy (TMA) is a rare and potentially life-threatening condition that can be caused by a heterogeneous group of diseases, often affecting the brain and kidneys. TMAs should be classified according to etiology to indicate targets for treatment. Complement dysregulation is an important cause of TMA that defines cases not related to coexisting conditions, that is, primary atypical hemolytic uremic syndrome (HUS). Ever since the approval of therapeutic complement inhibition, the approach of TMA has focused on the recognition of primary atypical HUS. Recent advances, however, demonstrated the pivotal role of complement dysregulation in specific subtypes of patients considered to have secondary atypical HUS. This is particularly the case in patients presenting with coexisting hypertensive emergency, pregnancy, and kidney transplantation, shifting the paradigm of disease. In contrast, complement dysregulation is uncommon in patients with other coexisting conditions, such as bacterial infection, drug use, cancer, and autoimmunity, among other disorders. In this review, we performed a critical appraisal on complement dysregulation and the use of therapeutic complement inhibition in TMAs associated with coexisting conditions and outline a pragmatic approach to diagnosis and treatment. For future studies, we advocate the term complement-mediated TMA as opposed to the traditional atypical HUS-type classification.

## 1. Integrated Discussion

**Figure 1:**
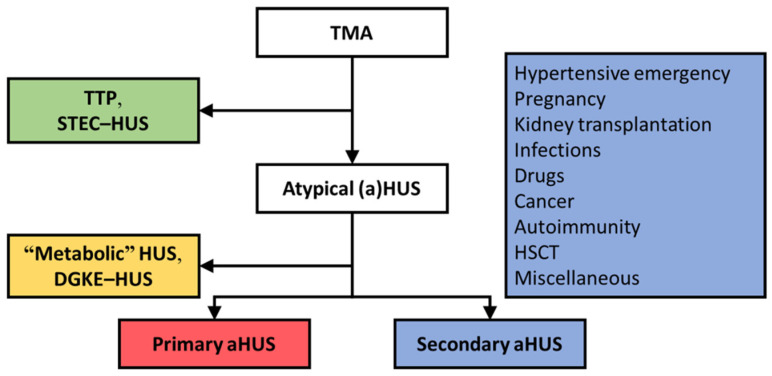
The atypical HUS-type classification [1,2].

Thrombotic microangiopathy (TMA) is a rare, potentially life-threatening condition that reflects tissue responses to severe endothelial damage caused by distinct disorders, including thrombotic thrombocytopenic purpura and hemolytic uremic syndrome (HUS). Despite heterogeneity, TMAs typically manifest with consumptive thrombocytopenia, microangiopathic hemolytic anemia, and ischemic organ damage, often affecting the brain and kidneys. TMAs should be classified according to etiology to indicate targets for treatment (Figure 1) [1,2]. For example, thrombotic thrombocytopenic purpura is caused by a severe deficiency of von Willebrand cleaving protease (also known as a disintegrin and metalloproteinase with thrombospondin type 1 motif, member 13 (ADAMTS13)) [3], and thus treatment should restore ADAMTS13’s function. The term HUS, either atypical or not, has been used to define *any* TMA with a normal functional activity of ADAMTS13.

HUS occurring on the background of complement dysregulation defines primary atypical HUS, indicating a diagnosis of exclusion [1]. Many of such patients present with rare variants in complement genes and/or autoantibodies that inhibit complement regulatory proteins [4,5]. Primary atypical HUS is considered an orphan disease, with an incidence of <1 per million population per year [6]. Most patients with HUS (i.e., ~90%) present with coexisting conditions, assumed to be the etiologic factor of disease, and have been termed secondary atypical HUS (Figure 1) [7]. Known coexisting conditions linked to secondary atypical HUS are hypertensive emergency, pregnancy, kidney transplantation, bacterial infections, drug use, cancer, autoimmunity, and hematologic stem cell transplantation (HSCT), among others. Recent advances, however, linked complement dysregulation to specific subtypes of so-called secondary atypical HUS and poor kidney outcomes [8,9,10]. Thus, the traditional atypical HUS-type classification is not absolute because complement dysregulation can be present along the spectrum of HUS [11]. In the era of therapeutic complement inhibition [12,13,14], the challenge is to recognize patients with complement dysregulation in the earliest possible stage to prevent end-stage kidney disease (ESKD).

In this review, we performed a critical appraisal on complement dysregulation and therapeutic complement inhibition in HUS presenting with coexisting conditions and outline a pragmatic approach to diagnosis and treatment. We advocate to use the term complement-mediated (C-)TMA to define cases related to complement dysregulation.

Secondary atypical HUS represents the majority of TMAs, that is, ~90%; Shiga toxin-producing *E. coli* (STEC)-HUS, thrombotic thrombocytopenic purpura (TTP), and primary atypical HUS are responsible for 6%, 3%, and 3% of TMAs [7]. DGKE, diacylglycerol kinase epsilon. HSCT, hematopoietic stem cell transplantation.

## 2. Primary Atypical HUS, a Prototypic C-TMA

**Figure 2:**
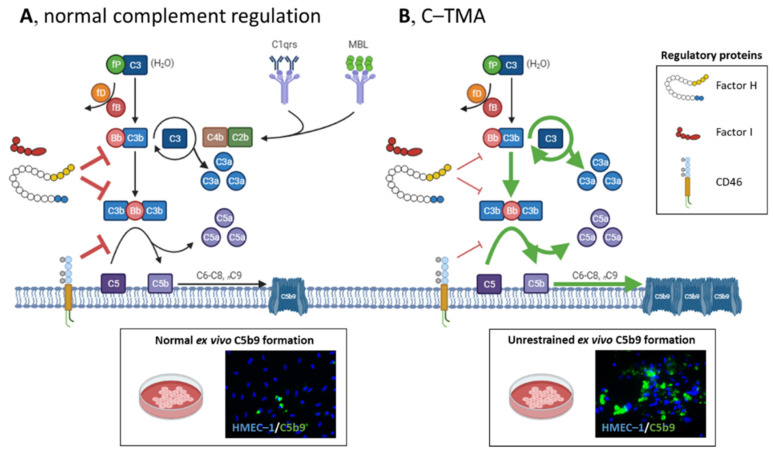
Schematic overview of complement activation and regulation in health and disease. (**A**) The complement system can be initiated via the classical (C1qrs), lectin (MBL), and alternative pathways (C3), converging to C3. The alternative pathway is a spontaneously and continuously active surveillance system operating in the circulation and on the cell surface. C3 (H_2_O) binds factor B (fB) and factor D (fD), and the latter cleaves fB into Bb, the serine esterase that cleaves C3 into C3a and C3b. C3’s thioester domain located in C3b can bind to the cell surface (e.g., microbes), providing a platform to form the C3 convertase of the alternative pathway (i.e., C3Bb) to cleave more C3, activating an amplification loop. Next, additional C3b can shift the C3 convertase to a C5 convertase, cleaving C5 into C5a and C5b, activating the terminal complement pathway. C5a and, to a lesser extent, C3a attract leukocytes to the site of complement activation. C5b can bind C6, C7, C8, and various C9 molecules to form the lytic C5b9 (i.e., membrane attack complex) on cells. Host cells, including the endothelium, are protected from the harmful effects of complement activation by factor I, factor H, and CD46 (also known as membrane cofactor protein); these proteins have decay-accelerating and cofactor activities, leading to factor I-mediated cleavage of C3b into inactivated proteins. (Normal ex vivo C5b9 formation on perturbed human microvascular endothelial cells of dermal origin (HMEC–1) indicates normal complement regulation.). (**B**) In C-TMA, rare variants in complement genes (i.e., loss of function of factor I, factor H, or CD46 (thin red lines); gain of function of C3 or CFB (green lines)) and/or autoantibodies targeting complement regulatory proteins result in unrestrained complement activation, formation of C5b9 on the endothelium, and a procoagulant environment that triggers thrombosis. (Massive ex vivo C5b9 formation on perturbed HMEC–1 indicates unrestrained C5 activation.) fP, properdin.

The complement system is an ancient and conserved effector system involved in the defense against pathogens and host homeostasis, which can be activated via the classic, lectin, and alternative pathways (Figure 2A). The alternative pathway is continuously active through a mechanism known as the thick-over, i.e., spontaneous hydrolysis of C3. Host cells, including the endothelium, are protected from the harmful effects of complement activation by regulatory proteins.

In the late 1980s, complement dysregulation (i.e., factor H deficiency) was found in two brothers with (primary atypical) HUS [15]. Thereafter, a linkage study in three families identified a variant in *CFH* [16] located in the C’-terminal-reduced factor H’s binding to the endothelium [17]. At present, >600 variants in complement genes have been identified in primary atypical HUS [18], with a prevalence of >50% [4,5]. Rare variants (i.e., minor allele frequency of <0.1%) [19] in *CFH*, *CFI*, *CD46*, *C3*, and *CFB* are of particular interest [18]. *CFH*, *CFI*, and *CD46* variants lead to impaired protein synthesis or function, whereas *C3* and *CFB* variants cause a gain-of-function protein, predisposing to unrestrained complement activation on the endothelium (Figure 2B). Recombination between *CFH* and the *CFH*-related genes *CFHR1*-*5* can form a hybrid gene linked to complement dysregulation, whereas single variants in *CFHR* genes require functional studies to determine their relevance [18]. The homozygous deletion of *CFHR1* and *CFHR3* has been associated with autoantibodies that prevent factor H’s binding to the endothelium [20,21]. The presence of such defects per se is insufficient to cause TMA [22], although combined variants increase the penetrance of disease [23]. Thus, additional precipitants, such as coexisting conditions, are needed for TMA to manifest.

In vivo studies, using factor H knockout mice [24] or mice homozygous for a gain-of-function change in *C3* [25], linked C5 activation to microvascular thrombosis. C5 activation on the endothelium leads to the expression and secretion of tissue factor via the insertion of sublytic C5b9 [26] and the interaction of C5a with its receptor [27]. Induction of tissue factor, activation of the extrinsic pathway of coagulation, and assembly of the prothrombinase complex cause fibrin thrombi to form. Of note, thrombin [28] and plasmin [29], upon activation of the fibrinolytic pathway, may accelerate C5 activation. In addition, C5 products cause the release of Weibel–Palade bodies, containing von Willebrand factor, from the endothelium [30], platelets, and leukocytes. This may provide another platform for thrombosis [31], but significant accumulation of von Willebrand factor multimers, as seen in thrombotic thrombocytopenic purpura, does not occur [32].

The introduction of eculizumab, an anti-C5 monoclonal antibody, improved 1-year kidney survival from 44% [5] to 90% [14]. Patients who start treatment early have the best possible chance to recover kidney function [13]. Kidney function further improved during extended treatment [12,13]. Ever since eculizumab’s success, several complement-specific drugs have reached late-stage clinical development for the treatment of primary atypical HUS.

Altogether, these experimental and clinical observations confirmed the role of complement in primary atypical HUS and changed the paradigm of disease.

## 3. Patients with TMA and Coexisting Conditions May Present with Complement Dysregulation

Recent studies demonstrated that complement dysregulation is prevalent in specific subtypes of “secondary” atypical HUS and linked to poor kidney outcomes, resembling primary atypical HUS (hereafter referred to as C-TMA) [33].

### 3.1. Hypertensive Emergency

Hypertensive emergency has been linked to activation of the renin–angiotensin system [34]. Renin can cause the C3 convertase to form via activation of C3 [35]. In vivo data showed that downstream activation of C5 may play a role in the development of hypertension-associated kidney disease [36,37].

Kidney disease is common (i.e., ~25%) in patients with hypertensive emergency and has been associated with hemolysis [38]. Patients with kidney disease are at risk for ESKD despite blood pressure control [39]. Many of such patients have been classified as “hypertensive” ESKD with no confirmative proof on kidney biopsy, assuming that the kidneys are the victim rather than culprit of disease. Thus, parenchymal kidney disease, including TMA, can be missed. This is particularly the case in patients without profound hematologic abnormalities [8]. We, for the first time, demonstrated the high prevalence of pathogenic variants in complement genes in patients with TMA and coexisting hypertensive emergency, which was associated with ESKD and TMA recurrence [40]. Our observations have been validated in independent cohorts, confirming C-TMA associated with complement gene variants in ~50% of patients with hypertensive emergency and severe kidney disease [41,42]. It remains unknown whether or not complement dysregulation plays a role in patients with mild-to-moderate kidney disease.

The effect of blood pressure control, the cornerstone of treatment, is limited in patients with hypertensive emergency and severe kidney disease [39]. The high prevalence of rare variants in complement genes and/or massive ex vivo C5b9 formation on the endothelium pointed to complement as a potential target for treatment [43]. Retrospective studies from France [41], Spain/Portugal [42], and our own group [8] included 29 patients with hypertensive emergency and severe kidney disease, including patients on dialysis, who had been treated with eculizumab (Table 1). At 12 months, a renal response was achieved in 21 (72%) patients, suggesting a benefit of treatment as compared to historical data [39]. Future prospective trials are warranted to test the hypothesis that therapeutic complement inhibition will improve the outcome of patients presenting with severe kidney disease. Furthermore, the predictive role of functional ex vivo complement measures [8] and pathologic features, such as chronic vascular and tubulointerstitial damage [44], should be addressed.

### 3.2. Pregnancy

TMA can develop in 1 per 25,000 births [45], the etiology of which varies from thrombotic thrombocytopenic purpura [46] to pregnancy-associated atypical HUS in late pregnancy and the postpartum period [47]. In an international cohort, pregnancy-associated atypical HUS resembled C-TMA based on the high incidence of ESKD and prevalence of complement gene variants, that is, 41% to 56% [9,48]. Pregnancy, indeed, has been linked to the first episode of primary atypical HUS in ~20% of women [47]. Rare variants in complement genes per se, however, cannot predict the risk of pregnancy-associated atypical HUS in a given pregnancy [49]. Pregnancy-associated atypical HUS often develops in the setting of coexisting conditions, such as preeclampsia and bleeding [49,50].

Preeclampsia and HELLP, both microangiopathies of late pregnancy, have been linked to complement activation but not to complement dysregulation. Variants in complement genes were found in up to 18% (*n*/*N* = 7/40) [51] and 38% (*n*/*N* = 9/24) [52,53], respectively. Most variants, however, should be classified as uncertain or no significance according to current standards and guidelines [54]. Patients typically present with mild kidney disease and are at low risk for ESKD [7]. Moreover, preeclampsia and HELLP can develop in pregnant women treated with eculizumab [55], suggesting a mechanism not related to C5 activation. Preeclampsia or HELLP, however, may mask pregnancy-associated atypical HUS when the kidney function does not improve after delivery.

Kidney Disease: Improving Global Outcomes advocated that patients with pregnancy-associated atypical HUS should be treated as C-TMA (Table 1) [2].

**Table 1:** The effects of therapeutic complement inhibition in patients with TMA and coexisting hypertensive emergency or pregnancy. Single case reports have not been included.

		Presentation		Genotyping		Outcome at 12 Months			
	Eculizumab, *n*/*N*	Creatinine, mg/dL	Dialysis (%)	Rare Variants (%)	Pathogenic (%)	Renal Response (%)	ESKD (%)	Death (%)	ESKD in Untreated Patients
**Hypertensive emergency**									
Combined data	29/122	Unknown	Unknown	14 (48)	Unknown	21 (72)	7 (24)	1 (3)	
Cavero et al. [42]	9/19	8 (IQR, 7–9)	8 (89)	5 (56)	3 (33)	7 (78)	2 (22)	0	60% at 1 year (*N* = 10)
El Karoui et al. [41]	13/76	Unknown	Unknown	7 (54)	Unknown	9 (69)	4 (31)	0	64% at 1 year (*N* = 61)
Timmermans et al. [8]	7/26	7 (IQR, 4–9)	4 (57)	2 (29)	2 (29)	5 (71)	1 (14)	1 (14)	75% at 1 year (*N* = 16) ^a^
**Pregnancy-associated atypical HUS**									
Combined data	17/116	Unknown	Unknown	7 (41)	Unknown	15 (88)	2 (25)	0	
Bruel et al. [9]	4/87	Unknown	Unknown	2 (50)	Unknown	3 (75)	1 (25)	0	49% at last follow-up (*N* = 71)
Huerta et al. [48]	10/22	4 (IQR, 3–5)	3 (30)	4 (40)	4 (40)	10 (100)	0	0	55% at last follow-up (*N* = 11)
Timmermans et al. [49]	3/7	5 (IQR, 4–6)	3 (100)	1 (33)	0	2 (67)	1 (33)	0	50% at last follow-up (*N* = 4)

^a^ Patients with follow-up <12 months were excluded. HUS, hemolytic uremic syndrome. ESKD, end-stage kidney disease. IQR, inter quartile range.

### 3.3. Kidney Transplantation

Activation of complement has been linked to various stages of kidney transplantation, including, but not limited to, organ preservation, reperfusion during surgery, and rejection [56].

TMA after kidney transplantation, both de novo and recurrent disease, has been linked to rare variants in complement genes in 29% (*n*/*N* = 7/24) [10] and 68% (*n*/*N* = 39/57) [57], respectively. The risk of TMA after kidney transplantation is >36 times higher in patients with C-TMA in the native kidney as compared to those with ESKD due to other causes [58] and is associated with the genetic fingerprint [57]. Of note, “hypertensive” ESKD was diagnosed prior to kidney donation in three recipients with de novo TMA who carried rare variants in complement genes (pathogenic, *n*/*N* = 2/3) [10]. Thus, C-TMA may be missed in the native kidneys, particularly in patients with a history of “hypertensive” ESKD [59], as discussed earlier. Most cases of de novo TMA in transplant recipients, however, are related to concurrent medications (e.g., calcineurin inhibition) [60] or antibody-mediated rejection [61]. The clinical course of both conditions is not consistent with complement dysregulation as 1-year graft survival was common [62,63]. The precise prevalence of rare variants in complement genes, however, has not been studied.

No data are available on eculizumab for the treatment of de novo TMA in transplant recipients, but eculizumab’s efficacy has been proven for C-TMA recurrence [64]. The graft’s capacity to recover is limited as compared to the native kidneys [65], favoring preemptive treatment in selected cases. Prophylaxis prevented C-TMA recurrence and improved graft survival in patients at moderate and high risk but should not be used in patients with a variant in *CD46* alone because the donor kidney does not express mutated CD46. TMA related to antibody-mediated rejection, characterized by C4d deposits in peritubular capillaries and donor-specific alloantibodies, often fails to respond to eculizumab [63]. C4d deposits reflect activation via the classical pathway, and therefore C1 inhibition upstream of C5 may be a better target for treatment.

## 4. TMAs Unrelated to Complement Dysregulation

The prevalence of complement gene variants in patients with secondary TMA equals controls (i.e., <10%) [66], and the standard of care, that is, treatment directed against the underlying cause, has improved the prognosis over recent decades [7]. Thus, clinical observations (i.e., genetics, clinical response to standard of care, and low risk of TMA recurrence) indicate normal complement regulation (Table 2).

### 4.1. Shiga Toxin-Producing E. coli (STEC) and Other Bacterial Infections

Shiga toxin, the causative factor of STEC-HUS, can be internalized to the renal endothelium’s cytosol via globotriaosyl ceramide. After internalization, activation of C3, either via the lectin [96] or alternative pathway [97], and glomerular thrombosis have been observed in mice.

Most patients with STEC-HUS or those with post-diarrheal HUS not related to STEC present with increased levels of plasma C5b9, while pathogenic variants in complement genes have been found in 2% (*n*/*N* = 3/125) and 3% (*n*/*N* = 1/33), respectively [67,68,69]. Although >50% of patients present with severe kidney disease, the clinical outcome appeared favorable, with rapid improvement in kidney function [67,70,98]. ESKD developed in one patient with a pathogenic variant in *CFH*, indicating C-TMA [99].

The 2011 STEC-HUS outbreak in Germany, the largest to date (HUS developed in 855 out of 3842 STEC-infected patients) [100], provided new data on the clinical course and effects of various treatment strategies, including therapeutic complement inhibition [70]. Eculizumab was used in patients with severe disease but appeared of no short-term clinical benefit as compared to matched controls with similar disease severity treated with plasma exchange [101]. Data of double-blind randomized controlled trials (NCT02205541, EudraCT2016-000997-39) that studied the efficacy of eculizumab for the treatment of STEC-HUS are awaited. The German data, however, corroborated previous observational studies [71,98], indicating that STEC-HUS is an acute but self-limiting disease.

Patients with pneumococcal HUS may present with rare variants in complement genes [73]. The clinical outcome appeared rather favorable as compared to C-TMA [74], and confirmative studies are therefore needed.

### 4.2. Drug-Induced TMA

Many drugs have been reported to cause drug-induced TMA, although causative associations are often lacking. The Oklahoma Registry and Blood Center of Wisconsin provided support for causal associations between specific drugs and TMA [102,103]. Quinine, either related to drug-dependent antibodies or not, was the most common cause of definite drug-induced TMA. These antibodies react with platelets and, in some instances, the endothelium [104]. Most patients showed a clinical response after discontinuing the drug, confirming a mechanistic link [66,79]. Of note, variants in complement genes were not found in patients with drug-induced TMA related to quinine [79]. Similar observations have been documented for proteasome inhibition [76], among other drugs. The observed efficacy of eculizumab in 12 patients with drug-induced TMA may therefore reflect the natural course of disease; none of the patients carried pathogenic variants in complement genes [77].

Monoclonal anti-vascular endothelial growth factor (VEGF) antibodies have been linked to TMA [105]. VEGF plays a role in the homeostasis of the glycocalyx and is therefore important to maintain the integrity of the glomerular endothelium [106]. VEGF inhibition attenuates factor H function on the endothelium [106] and causes chronic rather than acute TMA, as is seen in C-TMA [105]. Indeed, no variants in *CFH*, *CFI*, and *CD46* were found in patients with TMA following anti-VEGF treatment [78].

No firm conclusions can be drawn on the role of complement in drug-induced TMA as drugs and mechanisms differ. C-TMA, however, is not anticipated in drug-induced TMA.

### 4.3. Cancer

TMA is a well-described complication of cancer, mostly linked to treatment (i.e., drug-induced TMA). The exact mechanism of cancer-related TMA is unknown, although cancer emboli and mucin produced by adenocarcinomas (e.g., gastric, lung, prostate) can damage the endothelium [107]. Most patients have mild-to-moderate kidney injury, pointing to normal complement regulation.

### 4.4. Autoimmunity

Defects in complement and clearance of apoptotic bodies are key for systemic lupus erythematosus to occur. Activation of complement, on the other hand, has been related to major organ involvement and, in particular, lupus nephritis [108]. TMA can be found in up to 20% of patients with lupus nephritis, mostly related to antiphospholipid antibodies [109]. In contrast to patients with lupus nephritis alone [110], those with lupus nephritis and TMA, either related to antiphospholipid antibodies or not, may present with variants of uncertain significance (*n*/*N* = 2/10) [18,54,84]. We found that patients with antiphospholipid syndrome unrelated to lupus nephritis and TMA on kidney biopsy have normal complement regulation. The prognosis of these patients is rather favorable as compared to those with C-TMA [81].

No data have been published on complement dysregulation in systemic sclerosis [111].

### 4.5. HSCT-TMA

TMA is a serious complication after HSCT, the incidence of which appeared to be 39% in a recent prospective study at the Cincinnati Children’s Hospital Medical Center [87], although lower rates have been reported. Patients with proteinuria and elevated serum C5b9 levels had a very poor prognosis, with death rates exceeding 80% at 12 months [87]. Homozygous deletions of *CFHR1* and *CFHR3*, either with factor H autoantibodies or not [112], have been found in a small subset of patients, while pathogenic variants in complement genes are uncommon [88,89]. The high incidence of HSCT-TMA as compared to C-TMA in the general population suggests that HSCT-TMA is not a simple Mendelian trait. HSCT-TMA has been associated with other factors, such as conditioning regimens, concurrent drugs, graft versus host disease, and infections. These factors may lower the threshold for TMA to manifest via neutrophils, neutrophil extracellular trap formation, and complement activation [113,114].

Remarkably, 64 patients with severe HSCT-TMA were treated with eculizumab for a median of 9 weeks, improving 1-year survival to 66% (*n*/*N* = 41/64) [88]. No relapse occurred ever since discontinuation. Currently, an open-label phase II study is enrolling patients to assess eculizumab’s efficacy (NCT03518203).

These observations linked (secondary) complement activation rather than dysregulation to the mechanism of HSCT-TMA.

### 4.6. Miscellaneous Conditions

TMA can also occur in relation to metabolic disorders (e.g., cobalamin deficiency) [94], loss of diacylglycerol kinase epsilon (DGKE) [90,93], and, perhaps, monoclonal gammopathies [95]. Metabolic disorders and loss of DGKE typically present in young children. Monoclonal gammopathies may be common in patients with TMA >50 years [95]. Additionally, TMA can occur in the postoperative period [115]. These TMAs do not usually develop on the background of complement dysregulation.

## 5. Proposal for a Pragmatic Approach to Diagnosis of TMA

With the current state of knowledge and availability of therapeutic complement inhibition, either eculizumab or other therapies under investigation, the central consideration in the management of patients with TMA is the recognition of C-TMA and, thus, patients who would likely benefit from such therapies.

**Figure 3:**
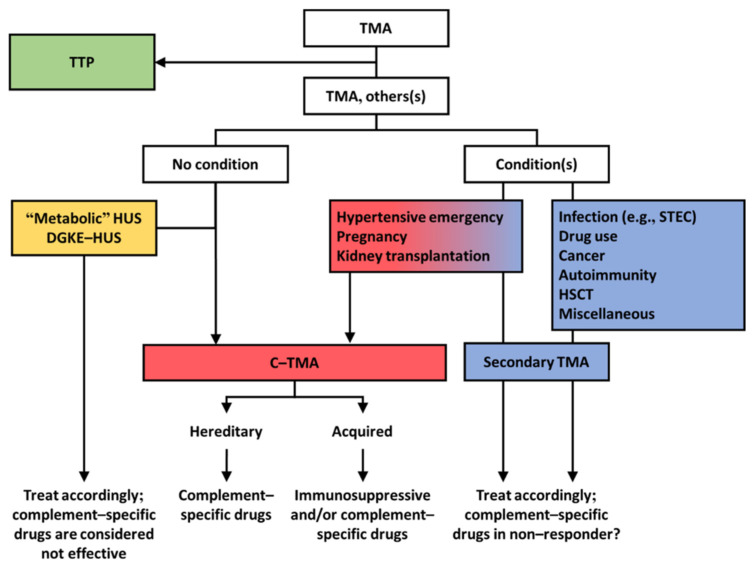
Pragmatic approach to diagnosis and treatment of TMA. Patients should be tested for the enzymatic activity of ADAMTS13 (i.e., >10% excludes thrombotic thrombocytopenic purpura (TTP)). Patients with a normal activity of ADAMTS13 should be screened for coexisting conditions. DGKE, diacylglycerol kinase epsilon. HSCT, hematopoietic stem cell transplantation. HUS, hemolytic uremic syndrome. STEC, Shiga toxin–producing *E. coli*.

**Figure 4:**
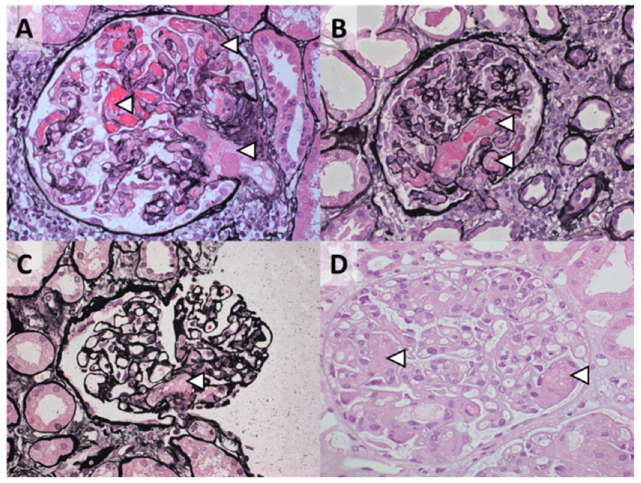
Morphologic features of TMA on kidney biopsy cannot define etiology. Representative cases of TMA presenting with coexisting hypertensive emergency: (**A**) 28-year-old woman with a gain-of-function C3 protein (p.R161W); (**B**) 47-year-old man with no rare variants in complement genes identified, after surgery; (**C**) 37-year-old woman with no rare variants in complement genes identified, and coexisting pregnancy; (**D**) 28-year-old woman with no rare variants in complement genes identified. The arrowheads indicate glomerular thrombosis, often accompanied by mesangiolysis. Jones methenamine silver (**A**–**C**) and hematoxylin and eosin (**D**) staining; original magnification, ×400.

We propose that TMAs presenting with a normal enzymatic activity of ADAMTS13 should be classified according to etiology (Figure 3). Profound systemic hemolysis can be absent [116], and therefore a tissue (e.g., kidney) biopsy may be needed to detect TMA. Morphologic features, however, cannot define etiology (Figure 4) [8]. Patients should be screened for coexisting conditions and, if absent, complement dysregulation. Patients with coexisting conditions and a severe clinical phenotype, that is, severe kidney disease not responding to the standard of care and/or TMA recurrence, should also be screened for complement dysregulation. Many patients with coexisting hypertensive emergency, pregnancy, and, to a lesser extent, de novo TMA after kidney transplantation fulfill these criteria and have C-TMA rather than secondary disease [11]. In contrast, C-TMA is uncommon in patients with bacterial infection, drug-induced TMA, cancer, autoimmunity, HSCT-TMA, and miscellaneous conditions, indicating secondary TMA; rapid improvement in kidney function should be expected in such cases (Table 2). TMA related to metabolic disorders (e.g., cobalamin C deficiency) or loss of DGKE is common in children and, in particular, infants. The term idiopathic should be used judiciously because the etiology can be found in almost every patient [7].

Of note, patients with coexisting hypertensive emergency, pregnancy, or de novo TMA after kidney transplantation may have C–TMA rather than secondary TMA. This, in particular, is the case in patients with severe kidney disease not responding to the standard of care and/or those with relapsing disease. Most patients with no coexisting conditions have C–TMA, although TMA related to recessive variants in *DGKE* and metabolic causes should be considered in children.

Tests recommended to screen for complement dysregulation include routine complement measures, genotyping, and autoantibody testing. Routine complement measures, however, are not specific and lack sensitivity [43,117]. Genetics should include sequencing of *CFH*, *CFI*, *CD46*, *CFB*, and *C3*, and multiplex ligation probe amplification to detect hybrid genes and/or the loss of *CFHR1* and *CFHR3*. Factor H serum reactivity should be assessed, particularly in children and patients with a homozygous deletion of *CFHR1* and *CFHR3* [20]. Hereditary and/or acquired factors inform the long-term prognosis [2] and should be used for classification (Figure 3) and to adopt suitable prophylactic measures [118]. For example, patients with pathogenic complement gene variants or high levels of factor H autoantibodies are at high risk of TMA recurrence and sequelae, contrasting patients with neither hereditary nor acquired factors. Functional assessment of ex vivo complement activation appears a promising method for the detection of unrestrained complement activation on the endothelium and, thus, C-TMA, irrespective of rare variants in complement genes [43,117]. Two functional tests have been developed using either microvascular endothelial cells of dermal origin (i.e., HMEC-1) [43,117] or endothelial hybrid cells that lack membrane-bound CD55 and CD59 (i.e., the modified Ham test) [119]. The HMEC-1 test reflects the dynamics of complement activation on the endothelium (Figure 2), with massive ex vivo C5b9 formation on resting endothelial cells at the time of active but not quiescent disease. The modified Ham test does not differentiate active from quiescent disease and lacks specificity [53]. Prospective studies are needed to test the hypothesis that functional tests can guide treatment decisions [33].

Rapid initiation of therapeutic complement inhibition is warranted in C-TMA, including patients with coexisting conditions. It remains unknown whether or not therapeutic complement inhibition should be used to treat specific subtypes of secondary TMA [77]. The efficacy of ravulizumab, a long-acting monoclonal antibody that blocks C5 activation, for the treatment of secondary TMA is being studied (NCT04743804). The results of this long-awaited randomized controlled trial will aid the debate of therapeutic complement inhibition in secondary TMA.

In conclusion, recent advances have clearly changed the landscape of TMAs. Knowledge on complement dysregulation has enabled breakthroughs in the diagnosis and treatment of C-TMA. The proposed approach will increase diagnosis and prognostic accuracy and thus may optimize the efficacy of treatment.

## Figures and Tables

**Table 2:** The prevalence of rare variants in complement genes (i.e., *CFH*, *CFI*, *CD46*, *CFB*, *C3*, *THBD*, and *CFHR1*-*CFHR5*) and disease course in patients with secondary TMA confirm normal complement regulation. Single case reports have not been included.

	Genetics/FHAA ^a^				Treatment	
Condition Ref. (Patients)	Rare Variants ^b^	Pathogenic	Dialysis at Presentation	Kidney Outcome ^c^	Standard of Care (SoC)	Eculizumab
**STEC-HUS**	9/125, 7%	3/125, 2%	>50%	1–7% ESKD; no relapse ^d^	Kidney response is common	Efficacy unproven
Ref. [67] (79)	6/75	3/75	56% (median, 9 d)	1% ESKD	Kidney response is common	Similar to SoC (*n* = 12)
Ref. [68] (25)	1/25	0/25	80% (mean, 7 d)	-	-	-
Ref. [69] (26)	2/25	0/25	65% (average, 16 d)	-	-	-
Ref. [70] (298)	-	-	54% (mean, 10 d)	1% ESKD	Kidney response is common	Similar to SoC (*n* = 67)
Ref. [71] (770)	-	-	57%	7% RRT at discharge	Kidney response is common	-
Ref. [72] (491)	-	-	57%	4% RRT at discharge	Kidney response is common	Similar to SoC (*n* = 193)
**Post-diarrheal HUS**						
Ref. [67] (33)	2/23, 9%	1/33, 3%	41% (median, 9 d)	0% ESKD; no relape	Kidney response is common	Efficayc unproven; similar to SoC (*n* = 3)
**Pneumococcal HUS**	3/5, 60%	2/5, 40%	>50%	14–23% ESKD; no relapse	Kidney response is common	Efficacy unproven
Ref. [73] (5)	3/5	2/5	80%	-	-	-
Ref. [74] (37)	–	–	73% (median, 15 d)	23% ESKD	Kidney response is common	-
Ref. [75] (14)	-	-	57%	14% RRT at discharge	Kidney response is common	-
**Drug-induced TMA**	2/62, 3%	1/62; 2%	Variable	<16% ESKD; no relapse ^d^	Kidney response is common	Efficacy unproven
Ref. [66] (32)	2/32	1/32	22%	16% ESKD	Kidney response is common	Similar to SoC (*n* = 13)
Ref. [76] (11)	0/2	0/2	45%	-	Kidney response is common	-
Ref. [77] (15)	0/15	0/15	33%	No ESKD	-	Kidney response is common
Ref. [78] (14)	0/13	0/13	-	No ESKD	Kidney response is common	-
Ref. [79] (19)	-	-	90%	17% ESKD	Kidney response is common	-
**Cancer**	2/11, 18%	1/11, 9%	Variable	ESKD competes with survival; no relapse	Kidney response is common	Efficacy unproven
Ref. [66] (11)	2/11	1/11	55%	30% ESKD	Kidney response is common	Similar to SoC (*n* = 8)
Ref. [80] (154)	-	-	17%	-	-	-
**Autoimmunity**	2/34, 6%	0/34, 0%	Variable	Variable: ~10% (i.e., CAPS) [81] to >50% ESKD [82]; no relapse	Poor prognosis	Efficacy unproven
Ref. [66] (26)	1/26	0/26	52%	61% ESKD	Poor prognosis	No response (*n*/*N* = 8/9)
Ref. [77] (8)	1/8	0/8	63%	57% ESKD	Poor prognosis	No response (*n*/*N* = 6/7)
Ref. [83] (10; CAPS)	1/10	0/10	-	-	-	-
Ref. [84] (11; SLE)	2/10	0/10	73%	27% ESKD	-	No response (*n*/*N* = 4/11)
**HSCT-TMA**	7/64, 11%	0/64, 0%	23%	Most surviving patients had CKD G3-4 [85,86]; no relapse	Poor prognosis with 1-year survival of 17% [87]	May be considered; 1-year survival of 66% [88]
Ref. [89] (34)	3/34	0/34	-	-	-	-
Ref. [88] (30)	4/30	0/30	23%	-	-	-
**DGKE-HUS**	3/72, 4%	0/72, 0%	50–69%	“late” ESKD; relapse is common	Kidney response is common	Efficacy unproven
Refs. [90,91] (44)	3/44	0/44	52%	23% ESKD (~12 yr); 70% relapse	Kidney response is common	Similar to SoC (*n* = 3)
Ref. [92] (15)	0/15	0/15	50% (i.e., <3 Wk)	13% ESKD (>20 yr); 53% relapse	Kidney response is common	Similar to SoC (*n* = 6)
Ref. [93] (13)	0/13	0/13	69%	31% ESKD (>10 yr); 77% relapse	Kidney response is common	Similar to SoC (*n* = 1)
**Cobalamine C deficiency**						
Ref. [94] (36)	2 ^e^/15, 13%	0/15, 0%	22%	High mortality; no relapse	Kidney response is common	Efficacy unproven; no response (*n*/*N* = 4/5)
**Monoclonal gammopathy**						
Ref. [95] (20)	-	-	55%	50% ESKD; no relapse	Kidney response is common	Efficacy unproven; no response (*n*/*N* = 1/1)

^a^ Rare variants indicate those with a minor allele frequency of <0.1%; detailed characteristics can be found in Table S1. ^b^ Number indicates pathogenic variants and variants of uncertain significance. ^c^ ESKD often within 1 year unless stated otherwise. ^d^ TMA recurrence linked to rare variant in complement genes. ^e^ Factor H autoantibodies were found in 1 patient with normal copies of CFHR1 and CFHR3, and thus these antibodies may not be relevant. CAPS, catastrophic antiphospholipid syndrome. CKD, chronic kidney disease (i.e., estimated GFR <60 mL/min/1.73 m^2^). D, days. DGKE, diacylglycerol kinase epsilon. TMA. ESKD, end-stage kidney disease. FHAA, factor H autoantibodies. HSCT, hematopoietic stem cell transplantation. SLE, systemic lupus erythematosus. STEC, Shiga toxin-producing *E. coli*. TMA, thrombotic microangiopathy. Wk, weeks.

## Data Availability

Not applicable.

## Supplementary Materials

The following are available online at https://www.mdpi.com/article/10.3390/jcm10143034/s1. Table S1. Classification of rare variants in *CFH*, *CFI*, *CD46*, *CFB*, *C3*, *THBD*, and *CFHR1*-*5* found in patients with secondary thrombotic microangiopathy.
