# Supplementary material for: The Syndromes of Thrombotic Microangiopathy: A Critical Appraisal on Complement Dysregulation
Source: J Clin Med. 2021 Jul 8;10(14):3034. doi: 10.3390/jcm10143034 (PMC8307963; doi:10.3390/jcm10143034)
Supplement: Supplementary file 1 [file jcm-10-03034-s001.zip › jcm-1258980-supplementary.pdf]

**Supplemental data**

# The Syndromes of Thrombotic Microangiopathy: A Critical Appraisal on Complement Dysregulation

**Sjoerd A. M. E. G. Timmermans [1,2,*] and Pieter van Paassen [1,2]**

[1] Department Nephrology and Clinical Immunology, Maastricht University Medical Center, 6229 HX Maastricht, The Netherlands; p.vanpaassen@maastrichtuniversity.nl
[2] Department Biochemistry, Cardiovascular Research Institute Maastricht (CARIM), 6229 HX Maastricht, The Netherlands
* Correspondence: sjoerd.timmermans@mumc.nl

**Table S1**. Classification of rare variants in *CFH*, *CFI*, *CD46*, *CFB*, *C3*, *THBD*, and *CFHR1-5* found in patients with secondary thrombotic microangiopathy.

| Condition | No. | Gene | cDNA | Protein | MAF (%) | PolyPhen-2/SIFT | *In vitro* | Significance[b] |
|---|---|---|---|---|---|---|---|---|
| **STEC-HUS** | | | | | | | | |
| Frémeaux-Bacchi *et al.*[1] (*N*=75) | 1 | *CFH* | c.2850G>T[c] | p.Q950H | 0.1-0.4 | Prob. damaging/Del. | LOF(?)[2] | Pathogenic → **Benign**[d] |
| | 2 | *THBD* | c.1483C>T[c] | p.P495S | 0.06-0.09 | Benign/Tol. | LOF[3] | **Pathogenic** |
| | 3 | *CFH* | c.1145C>A | p.A382E | 0 | Prob. damaging/Tol. | LOF[1] | **Pathogenic** |
| | 4 | *CD46* | c.503_504insA | p.N170Kfs*7 | 0 | N/a | LOF[1] | **Pathogenic** |
| | ? | *C3* | c.2203C>T[c] | p.R735W | 0.1-0.3 | Prob. damaging/Del. | Normal[4] | VUS → **Benign**[d] |
| | ? | *C3* | c.2203C>T[c] | p.R735W | 0.1-0.3 | Prob. damaging/Del. | Normal[4] | VUS → **Benign**[d] |
| | ? | *C3* | c.2203C>T[c] | p.R735W | 0.1-0.3 | Prob. damaging/Del. | Normal[4] | VUS → **Benign**[d] |
| | ? | *C3* | c.4369G>C | p.D1457H | 0.1 | Prob. damaging/Del. | – | VUS → **Benign**[d] |
| | ? | *C3* | c.1618G>T | p.A540S | <0.01 | Benign/Tol. | – | **VUS** |
| | ? | *CFB* | c.978A>C[c] | p.E326D | <0.1 | Benign/Tol. | – | **VUS** |
| | ? | *CFI* | c.782G>A[c] | p.G261D | 0.1 | Benign/Tol. | Normal[5] | VUS → **Benign**[d] |
| | ? | *THBD* | c.829G>T | p.G277W | 0 | Prob. damaging/Del. | – | **VUS** |
| Ahlenstiel-Grunow *et al.*[6] (*N*=25) | ? | *C3* | c.476T>A | p.V159E | 0 | Poss. damaging/Tol. | – | **VUS** |
| | ? | *CFI* | c.1657C>T[c] | p.P553S | 0.1 | Benign/Tol. | – | VUS → **Benign**[d] |
| Westra et al.[7] (*N*=25) | P14 | *C3* | c.3656G>A[c] | p.R1219H | <0.01 | Benign/Tol. | – | **VUS** |
| | P21 | *CFH* | c.2867C>T[c] | p.T956M | 0.1 | Poss. damaging/Tol. | Normal[8] | **Benign** |
| | P25 | *C3* | c.463T>G[c] | p.K155Q | 02.-0.4 | Benign/Tol. | GOF[9] | Pathogenic → **Benign**[d] |
| | P29 | *CFH* | c.172T>G[c] | p.S58A | 0.02 | Benign/Tol. | LOF[8] | **VUS** |
| Total, *n*/*N*[a] | 9/125 | | | | | | | **Pathogenic**, *n*/*N*=3/125 |
| **Post-diarrheal HUS** | | | | | | | | |
| Frémeaux-Bacchi *et al.*[1] (*N*=33) | 5 | *THBD* | c.127G>A[c] | p.A43T | 0.2-0.4 | Benign/Tol. | LOF[3] | Pathogenic → **Benign**[d] |
| | 6 | *CFH* | c.3628C>T[c] | p.R1210C | 0.02 | Benign/Tol. | LOF[10] | **Pathogenic** |
| | | *C3* | c.4319A>C[c] | p.D1440A | 0.02-0.05 | Benign/Tol. | – | **VUS** |
| | ? | *CFH* | c.2867C>T[c] | p.T956M | 0.1 | Poss. damaging/Tol. | Normal[8] | VUS → **Benign**[d] |
| | ? | *C3* | c.4855A>C[c] | p.S1619R | 0.1-0.2 | Poss. damaging/Tol. | Normal[11] | VUS → **Benign**[d] |
| | ? | *C3* | c.4855A>C[c] | p.S1619R | 0.1-0.2 | Poss. damaging/Tol. | Normal[11] | VUS → **Benign**[d] |
| | ? | *C3* | c.4177C>T | p.R1393W | <0.01 | Poss. damaging/Del. | – | **VUS** |
| Total, *n*/*N*[a] | 2/33 | | | | | | | **Pathogenic**, *n*/*N*=1/33 |
| **Pneumococcal HUS** | | | | | | | | |
| Szilágyi *et al.*[12] (*N*=5) | 3 | *CFH* | c.3445C>T | p.R1149X | <0.01 | N/a | – | **Pathogenic** |
| | 4 | *CFI* | c.148C>G[c] | p.P50A | <0.02 | Prob. damaging/Del. | LOF[13] | **Pathogenic** |
| | 5 | *THBD* | c.131C>T | p.T44I | <0.01 | Benign/Tol. | – | **VUS** |
| Total, *n*/*N*[a] | 3/5 | | | | | | | **Pathogenic**, *n*/*N*=2/5 |

| | | | | | | | | |
|---|---|---|---|---|---|---|---|---|
| **Drug-induced TMA** | | | | | | | | |
| Le Clech *et al.*[14] (*N*=32) | 3 | *CFI* | c.11T>A | p.L4H | 0.02 | Benign/Tol. | – | **VUS** |
| | 6 | *CFH* | c.3596T>C[c] | p.F1199S | 0 | Poss. damaging/Del. | LOF[15] | **Pathogenic** |
| Total, *n*/*N*[a] | 2/32 | | | | | | | **Pathogenic**, *n*/*N*=1/32 |
| **Cancer** | | | | | | | | |
| Le Clech *et al.*[14] (*N*=11) | 2 | *THBD* | c.91G>A | p.V31I | 0 | Benign/Tol. | – | **VUS** |
| | 5 | *CFH* | c.3047A>G[c] | p.Y1016C | 0 | Prob. damaging/Del. | – | **Pathogenic** |
| Total, *n*/*N*[a] | 2/11 | | | | | | | **Pathogenic**, *n*/*N*=1/11 |
| **Autoimmunity** | | | | | | | | |
| Le Clech *et al.*[14] (*N*=26) | 1 | *THBD* | c.707C>G | p.A236G | 0.02 | Benign/Tol. | – | **VUS** |
| Cavero *et al.*[16] (*N*=8) | 22 | *CFHR1* | c.869T>C | p.L290S | <0.01 | Benign/Tol. | – | Pathogenic → **VUS**[d] |
| | | *CFHR1* | c.887C>T | p.A296V | <0.1 | Benign/Tol. | – | Pathogenic → **VUS**[d] |
| Chaturverdi *et al.*[17] (*N*=10) | CAPS3 | *THBD* | c.1502C>T[c] | p.P501L | 0.2 | Benign/Del. | LOF[3] | Pathogenic → **Benign**[d] |
| | CAPS6 | *CFHR4* | c.860G>A | p.R287H | <0.01 | Benign/Tol. | – | **VUS** |
| Park *et al.*[18] (*N*=10) | 1 | *THBD* | c.127G>A[c] | p.A43T | 0.2-0.4 | Benign/Tol. | LOF[3] | **Benign** |
| | 2 | *CFH* | c.184G>A[c] | p.V62I | >5 | Benign/Tol. | – | **Benign** |
| | | *CFH* | c.1204C>T[c] | p.H402Y | >5 | Benign/Tol. | – | **Benign** |
| | 6 | *CFB* | c.724A>C[c] | p.I242L | <0.2 | Benign/Tol. | – | **VUS** |
| | | *CFHR5* | c.384G>T | p.S128S | 0.1 | Synonymous | – | **Benign** |
| | | *CD46* | c.989-78G>A | N/a | 35 | N/a | – | **Benign** |
| | | *CFH* | c.1204C>T[c] | p.H402Y | >5 | Benign/Tol. | – | **Benign** |
| | 8 | *THBD* | c.1456G>T[c] | p.D486Y | 0.02-0.2 | Benign/Tol. | LOF[3] | **VUS** |
| | | *CFH* | c.184G>A[c] | p.V62I | >5 | Benign/Tol. | – | **Benign** |
| | 9 | *CD46* | c.989-78G>A | N/a | 35 | N/a | – | **Benign** |
| | 11 | *CFI* | c.1217G>A | p.R406H | 0.1-1 | Benign/Tol. | Normal[19] | **Benign** |
| Total, *n*/*N*[a] | 5/54 | | | | | | | **Pathogenic**, *n*/*N*=0/54 |
| **HSCT-TMA** | | | | | | | | |
| Jodele *et al.*[20] (*N*=34) | ? | *CD46* | c.971C>T[c] | p.P324L | 0.2 | Prob. damaging/Del. | LOF[21] | VUS → **Benign**[d] |
| | ? | *CFI* | c.1246A>C[c] | p.I416L | 0.4 | Benign/Tol. | LOF[13] | Pathogenic → **Benign**[d] |
| | ? | *CD46* | c.796G>A[c] | p.D266N | 0.3-0.4 | Benign/Tol. | – | VUS → **Benign**[d] |
| | ? | *C3* | c.1243C>A | p.P415T | 0 | Benign/Tol. | – | **VUS** |
| | ? | *THBD* | c.1208G>A[c] | p.R403K | 0.02-0.03 | Benign/Tol. | – | **VUS** |
| | | *CFHR5* | c.486_487insA | p.E163Rfs*35 | 0.2 | N/a | – | Pathogenic → **Benign**[d] |
| | | *CFHR5* | c.622T>C | p.C208R | 0.2 | Prob. damaging/Del. | – | VUS → **Benign**[d] |
| | ? | *CFI* | c.1217G>A | p.R406H | 0.1-1 | Benign/Tol. | Normal[19] | VUS → **Benign**[d] |
| | ? | *C3* | c.463T>G[c] | p.K155Q | 0.2-0.4 | Benign/Tol. | GOF[9] | Pathogenic → **Benign**[d] |
| | ? | *CFI* | c.1534+5G>T[c] | N/a | 0.9-1.1 | N/a | – | Pathogenic → **Benign**[d] |
| | ? | *CFI* | c.1534+5G>T[c] | N/a | 0.9-1.1 | N/a | – | Pathogenic → **Benign**[d] |

| | | | | | | | | |
|---|---|---|---|---|---|---|---|---|
| | | *CFI* | c.1657C>T[c] | p.P553S | 0.1 | Benign/Tol. | – | VUS → **Benign**[d] |
| | ? | *CFI* | c.1322A>G[c] | p.K441R | 0.1 | Benign/Tol. | – | Pathogenic → **Benign**[d] |
| | | *CFB* | c.1697A>C[c] | p.E566A | 0.7 | Benign/Tol. | – | VUS → **Benign**[d] |
| | ? | *CFB* | c.1729G>A[c] | p.V577I | 0.01 | Benign/Tol. | – | **VUS** |
| | | *CFB* | c.2005G>C[c] | p.V669L | 0.01 | Benign/Tol. | – | **VUS** |
| Jodele *et al.*[22] (*N*=30) | 1 | *C3* | c.2203C>T[c] | p.R735W | 0.2-0.3 | Prob. damaging/Del. | Normal[4] | Pathogenic → **Benign**[d] |
| | | *CFHR3* | c.839_840del | p.I280fs | 0.1 | N/a | – | VUS → **Benign**[d] |
| | 2 | *CFB* | c.95G>A | p.R32Q | >5 | Benign/Tol. | – | VUS → **Benign**[d] |
| | | *CFHR3* | c.786A>T | p.P262P | >5 | N/a | – | VUS → **Benign**[d] |
| | 3 | *CFB* | c.95G>A | p.R32Q | >5 | Benign/Tol. | – | VUS → **Benign**[d] |
| | 4 | *CFHR5* | c.486_487insAA | p.E163fs | 0 | N/a. | – | **VUS** |
| | 5 | *CFB* | c.95G>A | p.R32Q | >5 | Benign/Tol. | – | VUS → **Benign**[d] |
| | | *CFH* | c.2850G>T[c] | p.Q950H | 0.1-0.4 | Prob. damaging/Del. | LOF(?)[2] | VUS → **Benign**[d] |
| | 6 | *THBD* | c.1502C>T[c] | p.P501L | 0.2 | Benign/Del. | LOF[3] | Pathogenic → **Benign**[d] |
| | 7 | *CFHR3* | c.786A>T | p.P262P | >5 | N/a | – | VUS → **Benign**[d] |
| | | *CFHR5* | c.1067G>A | p.R356H | >2 | Poss. damaging/Del. | – | **Benign** |
| | 8 | *CFB* | c.95G>A | p.R32Q | >5 | Benign/Tol. | – | VUS → **Benign**[d] |
| | 11 | *CFB* | c.559G>A | p.V187I | 0 | Poss. damaging/Del. | – | **VUS** |
| | | *CD46* | c.1058C>T[c] | p.A353V | >1 | Benign/Tol. | Normal[23] | VUS → **Benign**[d] |
| | 13 | *CFB* | c.1697A>C[c] | p.E556A | 0.7 | Benign/Tol. | – | VUS → **Benign**[d] |
| | 14 | *CFB* | c.1697A>C[c] | p.E556A | 0.7 | Benign/Tol. | – | VUS → **Benign**[d] |
| | | *CFHR1* | c.310C>T | p.H104Y | <0.03 | Benign/Tol. | – | **VUS** |
| | 18 | *CFB* | c.1697A>C[c] | p.E556A | 0.7 | Benign/Tol. | – | VUS → **Benign**[d] |
| | | *CFH* | c.3506T>C | p.I1169T | <0.01 | Benign/Del. | – | **VUS** |
| Total, *n*/*N*[a] | 7/64 | | | | | | | **Pathogenic**, *n*/*N*=0/64 |
| **DGKE-HUS** | | | | | | | | |
| Azukaitis *et al.*[24] (*N*=44) | HUS39 | *THBD* | c.1456G>T[c] | p.D486Y | 0.02-0.2 | Benign/Tol. | LOF[3] | Pathogenic → **VUS**[d] |
| | HUS40 | *THBD* | c.1456G>T[c] | p.D486Y | 0.02-0.2 | Benign/Tol. | LOF[3] | Pathogenic → **VUS**[d] |
| | HUS272 | *C3* | c.784G>T[c] | p.G262W | 0 | Prob. damaging/Del. | – | **VUS** |
| Total, *n*/*N*[a] | 3/44 | | | | | | | **Pathogenic**, *n*/*N*=0/44 |
| **Cobalamin C deficiency** | | | | | | | | |
| Beck *et al.*[25] | 27 | *CFH* | Unknown | N/a | N/a | N/a | N/a | **VUS** |
| Total, *n*/*N*[a] | 1/27 | | | | | | | **Pathogenic**, *n*/*N*=0/27 |

[a] Number indicates pathogenic variants and variants of uncertain significance.

[b] The classification of variants was based on international standards.[26, 27] Pathogenic variants (depicted in red) were defined as those with functional studies supporting a defect in complement regulation, including null variants in genes linked to complement regulation (not including *CFB* and *C3*) and at least one of the following: located in a mutational hotspot, located in a functional domain, and/or cluster in patients with

primary atypical hemolytic uremic syndrome.[27] Benign variants (depicted in green) were defined as those with a minor allele frequency of ≥0.1% and/or functional studies supporting normal complement regulation. Rare variants, that is, those with a minor allele frequency of <0.1%, not fulfilling these criteria have been classified as uncertain significance.

[c] Variant has been identified in the database of complement gene variants (http://www.complement-db.org/home.php).

[d] Variant has been reclassified.

Del., deleterious. DGKE, diacylglycerol kinase epsilon. GOF, gain-of-function. HSCT-TMA, hematopoietic stem cell transplantation-thrombotic microangiopathy. HUS, hemolytic uremic syndrome. LOF, loss-of-function. MAF, minor allele frequency according to the Exome Variant Server (EVS) and Genome Aggregation Database (gnomAD). Prob. damaging, probably damaging. Poss. damaging, possibly damaging. STEC, Shiga toxin-producing E. *coli*. Tol., tolerated. VUS, variant of uncertain significance.

# References


1. Fremeaux-Bacchi, V.; Sellier-Leclerc, A. L.; Vieira-Martins, P.; Limou, S.; Kwon, T.; Lahoche, A.; Novo, R.; Llanas, B.; Nobili, F.; Roussey, G.; Cailliez, M.; Ulinski, T.; Deschenes, G.; Alberti, C.; Weill, F. X.; Mariani, P.; Loirat, C., Complement Gene Variants and Shiga Toxin-Producing Escherichia coli-Associated Hemolytic Uremic Syndrome: Retrospective Genetic and Clinical Study. *Clin J Am Soc Nephrol* **2019,** *14* (3), 364-377.
2. Mohlin, F. C.; Nilsson, S. C.; Levart, T. K.; Golubovic, E.; Rusai, K.; Muller-Sacherer, T.; Arbeiter, K.; Pallinger, E.; Szarvas, N.; Csuka, D.; Szilagyi, A.; Villoutreix, B. O.; Prohaszka, Z.; Blom, A. M., Functional characterization of two novel non-synonymous alterations in CD46 and a Q950H change in factor H found in atypical hemolytic uremic syndrome patients. *Mol Immunol* **2015,** *65* (2), 367-76.
3. Delvaeye, M.; Noris, M.; De Vriese, A.; Esmon, C. T.; Esmon, N. L.; Ferrell, G.; Del-Favero, J.; Plaisance, S.; Claes, B.; Lambrechts, D.; Zoja, C.; Remuzzi, G.; Conway, E. M., Thrombomodulin mutations in atypical hemolytic-uremic syndrome. *N Engl J Med* **2009,** *361* (4), 345-57.
4. Fremeaux-Bacchi, V.; Miller, E. C.; Liszewski, M. K.; Strain, L.; Blouin, J.; Brown, A. L.; Moghal, N.; Kaplan, B. S.; Weiss, R. A.; Lhotta, K.; Kapur, G.; Mattoo, T.; Nivet, H.; Wong, W.; Gie, S.; Hurault de Ligny, B.; Fischbach, M.; Gupta, R.; Hauhart, R.; Meunier, V.; Loirat, C.; Dragon-Durey, M. A.; Fridman, W. H.; Janssen, B. J.; Goodship, T. H.; Atkinson, J. P., Mutations in complement C3 predispose to development of atypical hemolytic uremic syndrome. *Blood* **2008,** *112* (13), 4948-52.
5. Nilsson, S. C.; Karpman, D.; Vaziri-Sani, F.; Kristoffersson, A. C.; Salomon, R.; Provot, F.; Fremeaux-Bacchi, V.; Trouw, L. A.; Blom, A. M., A mutation in factor I that is associated with atypical hemolytic uremic syndrome does not affect the function of factor I in complement regulation. *Mol Immunol* **2007,** *44* (8), 1835-44.
6. Ahlenstiel-Grunow, T.; Hachmeister, S.; Bange, F. C.; Wehling, C.; Kirschfink, M.; Bergmann, C.; Pape, L., Systemic complement activation and complement gene analysis in enterohaemorrhagic Escherichia coli-associated paediatric haemolytic uraemic syndrome. *Nephrol Dial Transplant* **2016,** *31* (7), 1114-21.
7. Westra, D.; Volokhina, E. B.; van der Molen, R. G.; van der Velden, T. J.; Jeronimus-Klaasen, A.; Goertz, J.; Gracchi, V.; Dorresteijn, E. M.; Bouts, A. H.; Keijzer-Veen, M. G.; van Wijk, J. A.; Bakker, J. A.; Roos, A.; van den Heuvel, L. P.; van de Kar, N. C., Serological and genetic complement alterations in infection-induced and complement-mediated hemolytic uremic syndrome. *Pediatr Nephrol* **2017,** *32* (2), 297-309.
8. Merinero, H. M.; Garcia, S. P.; Garcia-Fernandez, J.; Arjona, E.; Tortajada, A.; Rodriguez de Cordoba, S., Complete functional characterization of disease-associated genetic variants in the complement factor H gene. *Kidney Int* **2018,** *93* (2), 470-481.
9. Seddon, J. M.; Yu, Y.; Miller, E. C.; Reynolds, R.; Tan, P. L.; Gowrisankar, S.; Goldstein, J. I.; Triebwasser, M.; Anderson, H. E.; Zerbib, J.; Kavanagh, D.; Souied, E.; Katsanis, N.; Daly, M. J.; Atkinson, J. P.; Raychaudhuri, S., Rare variants in CFI, C3 and C9 are associated with high risk of advanced age-related macular degeneration. *Nat Genet* **2013,** *45* (11), 1366-70.
10. Ferreira, V. P.; Herbert, A. P.; Cortes, C.; McKee, K. A.; Blaum, B. S.; Esswein, S. T.; Uhrin, D.; Barlow, P. N.; Pangburn, M. K.; Kavanagh, D., The binding of factor H to a complex of physiological polyanions and C3b on cells is impaired in atypical hemolytic uremic syndrome. *J Immunol* **2009,** *182* (11), 7009-18.
11. Mohlin, F. C.; Gros, P.; Mercier, E.; Gris, J. R.; Blom, A. M., Analysis of C3 Gene Variants in Patients With Idiopathic Recurrent Spontaneous Pregnancy Loss. *Front Immunol* **2018,** *9*, 1813.
12. Szilagyi, A.; Kiss, N.; Bereczki, C.; Talosi, G.; Racz, K.; Turi, S.; Gyorke, Z.; Simon, E.; Horvath, E.; Kelen, K.; Reusz, G. S.; Szabo, A. J.; Tulassay, T.; Prohaszka, Z., The role of complement in Streptococcus pneumoniae-associated haemolytic uraemic syndrome. *Nephrol Dial Transplant* **2013,** *28* (9), 2237-45.
13. Bienaime, F.; Dragon-Durey, M. A.; Regnier, C. H.; Nilsson, S. C.; Kwan, W. H.; Blouin, J.; Jablonski, M.; Renault, N.; Rameix-Welti, M. A.; Loirat, C.; Sautes-Fridman, C.; Villoutreix, B. O.; Blom, A. M.; Fremeaux-Bacchi, V., Mutations in components of complement influence the outcome of Factor I-associated atypical hemolytic uremic syndrome. *Kidney Int* **2010,** *77* (4), 339-49.
14. Le Clech, A.; Simon-Tillaux, N.; Provot, F.; Delmas, Y.; Vieira-Martins, P.; Limou, S.; Halimi, J. M.; Le Quintrec, M.; Lebourg, L.; Grange, S.; Karras, A.; Ribes, D.; Jourde-Chiche, N.; Rondeau, E.; Fremeaux-Bacchi, V.; Fakhouri, F., Atypical and secondary hemolytic uremic syndromes have a distinct presentation and no common genetic risk factors. *Kidney Int* **2019,** *95* (6), 1443-1452.
15. Dragon-Durey, M. A.; Fremeaux-Bacchi, V.; Loirat, C.; Blouin, J.; Niaudet, P.; Deschenes, G.; Coppo, P.; Herman Fridman, W.; Weiss, L., Heterozygous and homozygous factor h deficiencies associated with hemolytic uremic syndrome or membranoproliferative glomerulonephritis: report and genetic analysis of 16 cases. *J Am Soc Nephrol* **2004,** *15* (3), 787-95.
16. Cavero, T.; Rabasco, C.; Lopez, A.; Roman, E.; Avila, A.; Sevillano, A.; Huerta, A.; Rojas-Rivera, J.; Fuentes, C.; Blasco, M.; Jarque, A.; Garcia, A.; Mendizabal, S.; Gavela, E.; Macia, M.; Quintana, L. F.; Maria Romera, A.; Borrego, J.; Arjona, E.; Espinosa, M.; Portoles, J.; Gracia-Iguacel, C.; Gonzalez-Parra, E.; Aljama, P.; Morales, E.; Cao, M.; Rodriguez de Cordoba, S.; Praga, M., Eculizumab in secondary atypical haemolytic uraemic syndrome. *Nephrol Dial Transplant* **2017,** *32* (3), 466-474.
17. Chaturvedi, S.; Braunstein, E. M.; Yuan, X.; Yu, J.; Alexander, A.; Chen, H.; Gavriilaki, E.; Alluri, R.; Streiff, M. B.; Petri, M.; Crowther, M. A.; McCrae, K. R.; Brodsky, R. A., Complement activity and complement regulatory gene mutations are associated with thrombosis in APS and CAPS. *Blood* **2020,** *135* (4), 239-251.
18. Park, M. H.; Caselman, N.; Ulmer, S.; Weitz, I. C., Complement-mediated thrombotic microangiopathy associated with lupus nephritis. *Blood Adv* **2018,** *2* (16), 2090-2094.
19. de Jong, S.; Volokhina, E. B.; de Breuk, A.; Nilsson, S. C.; de Jong, E. K.; van der Kar, N.; Bakker, B.; Hoyng, C. B.; van den Heuvel, L. P.; Blom, A. M.; den Hollander, A. I., Effect of rare coding variants in the CFI gene on Factor I expression levels. *Hum Mol Genet* **2020,** *29* (14), 2313-2324.
20. Jodele, S.; Zhang, K.; Zou, F.; Laskin, B.; Dandoy, C. E.; Myers, K. C.; Lane, A.; Meller, J.; Medvedovic, M.; Chen, J.; Davies, S. M., The genetic fingerprint of susceptibility for transplant-associated thrombotic microangiopathy. *Blood* **2016,** *127* (8), 989-96.
21. Mohlin, F. C.; Mercier, E.; Fremeaux-Bacchi, V.; Liszewski, M. K.; Atkinson, J. P.; Gris, J. C.; Blom, A. M., Analysis of genes coding for CD46, CD55, and C4b-binding protein in patients with idiopathic, recurrent, spontaneous pregnancy loss. *Eur J Immunol* **2013,** *43* (6), 1617-29.
22. Jodele, S.; Dandoy, C. E.; Lane, A.; Laskin, B. L.; Teusink-Cross, A.; Myers, K. C.; Wallace, G.; Nelson, A.; Bleesing, J.; Chima, R. S.; Hirsch, R.; Ryan, T. D.; Benoit, S.; Mizuno, K.; Warren, M.; Davies, S. M., Complement blockade for TA-TMA: lessons learned from a large pediatric cohort treated with eculizumab. *Blood* **2020,** *135* (13), 1049-1057.
23. Caprioli, J.; Noris, M.; Brioschi, S.; Pianetti, G.; Castelletti, F.; Bettinaglio, P.; Mele, C.; Bresin, E.; Cassis, L.; Gamba, S.; Porrati, F.; Bucchioni, S.; Monteferrante, G.; Fang, C. J.; Liszewski, M. K.; Kavanagh, D.; Atkinson, J. P.; Remuzzi, G.; International Registry of, R.; Familial, H. T., Genetics of HUS: the impact of MCP, CFH, and IF mutations on clinical presentation, response to treatment, and outcome. *Blood* **2006,** *108* (4), 1267-79.
24. Azukaitis, K.; Simkova, E.; Majid, M. A.; Galiano, M.; Benz, K.; Amann, K.; Bockmeyer, C.; Gajjar, R.; Meyers, K. E.; Cheong, H. I.; Lange-Sperandio, B.; Jungraithmayr, T.; Fremeaux-Bacchi, V.; Bergmann, C.; Bereczki, C.; Miklaszewska, M.; Csuka, D.;

Prohaszka, Z.; Killen, P.; Gipson, P.; Sampson, M. G.; Lemaire, M.; Schaefer, F., The Phenotypic Spectrum of Nephropathies Associated with Mutations in Diacylglycerol Kinase epsilon. *J Am Soc Nephrol* **2017,** *28* (10), 3066-3075.

25. Beck, B. B.; van Spronsen, F.; Diepstra, A.; Berger, R. M.; Komhoff, M., Renal thrombotic microangiopathy in patients with cblC defect: review of an under-recognized entity. *Pediatr Nephrol* **2017,** *32* (5), 733-741.
26. Richards, S.; Aziz, N.; Bale, S.; Bick, D.; Das, S.; Gastier-Foster, J.; Grody, W. W.; Hegde, M.; Lyon, E.; Spector, E.; Voelkerding, K.; Rehm, H. L.; Committee, A. L. Q. A., Standards and guidelines for the interpretation of sequence variants: a joint consensus recommendation of the American College of Medical Genetics and Genomics and the Association for Molecular Pathology. *Genet Med* **2015,** *17* (5), 405-24.
27. Osborne, A. J.; Breno, M.; Borsa, N. G.; Bu, F.; Fremeaux-Bacchi, V.; Gale, D. P.; van den Heuvel, L. P.; Kavanagh, D.; Noris, M.; Pinto, S.; Rallapalli, P. M.; Remuzzi, G.; Rodriguez de Cordoba, S.; Ruiz, A.; Smith, R. J. H.; Vieira-Martins, P.; Volokhina, E.; Wilson, V.; Goodship, T. H. J.; Perkins, S. J., Statistical Validation of Rare Complement Variants Provides Insights into the Molecular Basis of Atypical Hemolytic Uremic Syndrome and C3 Glomerulopathy. *J Immunol* **2018,** *200* (7), 2464-2478.
